# Current status and reflections on the diagnosis and treatment of respiratory tract infections in children in the COVID‐19 pandemic and post‐COVID‐19 era

**DOI:** 10.1002/pdi3.33

**Published:** 2023-10-14

**Authors:** Yuyi Tang, Luo Ren, Enmei Liu

**Affiliations:** ^1^ Department of Respiratory Medicine Children's Hospital of Chongqing Medical University National Clinical Research Center for Child Health and Disorders Ministry of Education Key Laboratory of Child Development and Disorders China International Science and Technology Cooperation Base of Child Development and Critical Disorders Chongqing Key Laboratory of Pediatrics Chongqing China; ^2^ Pediatric Research Institute Children's Hospital of Chongqing Medical University Chongqing China

**Keywords:** children, COVID‐19, non‐pharmaceutical interventions, post‐COVID‐19 era, respiratory virus

## Abstract

Respiratory tract infections (RTIs) are common and frequently occurring diseases in children, posing a significant health threat to children worldwide. Viruses are the most important pathogens of childhood RTIs. Since the outbreak of Coronavirus Disease 2019 (COVID‐19), a series of nonpharmaceutical interventions (NPIs) have been widely implemented around the globe, and important changes have taken place in the spectrum of respiratory diseases and viruses in children. However, with relaxation of NPIs, there has been a “virus resurgence” in some areas, with multiple viral infectious diseases appearing simultaneously. This review comprehensively summarizes the changes observed in the spectrum of respiratory diseases and viruses in children in the context of the COVID‐19 pandemic, explores possible mechanisms, and presents reflections on the key points of diagnosis and treatment of RTIs in children in the post‐COVID‐19 era in light of recent advances in COVID‐19 in children.

## 1 INTRODUCTION

Respiratory tract infections (RTIs) pose a serious threat to children's health and are the leading cause of death in children under 5 years of age worldwide. Viruses are the main pathogen of RTIs in children. Globally, one or more respiratory viruses are detected in 55% of children under 18 years of age with community‐acquired pneumonia.^1^ A study by the Chinese Center for Disease Control and Prevention also reported 46.9% of virus detection in children under 5 years of age with RTIs.^2^

On March 12, 2020, the World Health Organization declared Coronavirus Disease 2019 (COVID‐19) a global pandemic.^3^ This new infectious disease caused by Severe Acute Respiratory Syndrome Coronavirus 2 (SARS‐CoV‐2) has brought great challenges to global public health security and medical service systems. Since the outbreak of COVID‐19, various nonpharmaceutical interventions (NPIs) targeting SARS‐CoV‐2 have been adopted due to the lack of specific antiviral medications and vaccines, and it appears that NPIs have also been successful in interfering with the spread of some non‐SARS‐CoV‐2 respiratory pathogens.

During the last 3 years of fighting against COVID‐19, China has strictly implemented NPIs and committed to vaccine and drug development. On January 8, 2023, China announced that the management of COVID‐19 was downgraded to the less strict Category B from previously top‐level Category A.

This review aims to summarize the changes in the spectrum of respiratory diseases and viruses in children in the context of COVID‐19, explore possible mechanisms, and discuss how SARS‐CoV‐2 and other respiratory viruses will coexist in the post‐pandemic era.

## 2 CHANGES IN THE SPECTRUM OF RESPIRATORY DISEASES DURING THE COVID‐19 PANDEMIC

Overall, during the COVID‐19 pandemic, there was a notable reduction in outpatient and hospitalization rates for children with non‐COVID‐19 diseases, especially RTIs, compared with the pre‐COVID‐19 period. However, the disease severity did not seem to change significantly. A multicenter cross‐sectional study revealed a 45.4% decrease in U.S. pediatric admissions in April 2020 compared with the previous decade.^4^ Similarly, in Beijing, China, outpatient visits for respiratory infectious diseases decreased by 51.8% and 58.4% in 2020 compared with 2018 and 2019, respectively. Unexpectedly, the number of outpatient visits for nonrespiratory infectious diseases exceeded that for respiratory infectious diseases in 2020.^5^ The number of cases of infectious diseases such as pneumonia, aseptic encephalitis, and sepsis admitted to the pediatric intensive care unit decreased in comparison to the same pre‐COVID‐19 period; however, noninfectious diseases, including trauma and poisoning, remained the main causes of critically ill children.^6^ Specifically, among childhood respiratory diseases, bronchiolitis, influenza, and asthma showed the most significant reductions.^7^, ^8^ Similar changes in the disease spectrum have been reported in the United Kingdom, Italy, Spain, Israel, etc.^9^, ^10^, ^11^, ^12^

## 3 CHANGES IN THE SPECTRUM OF RESPIRATORY VIRUSES DURING THE COVID‐19 PANDEMIC

During COVID‐19, the activity of most common respiratory viruses in children was reduced compared with that during pre‐COVID‐19. Nevertheless, a few viruses remained active. Subsequent to relaxation of NPIs, the detection rate of some viruses increased sharply, displaying atypical surges and seasonality shifts. Variations in viral dynamics were observed across diverse regions, populations, and time frames under investigation.

### 3.1 Human respiratory syncytial virus

Human respiratory syncytial virus (HRSV) is a common and highly contagious virus of RTIs in infants. The newly published systematic review of the burden of HRSV showed that there were 33 million episodes of HRSV‐associated acute lower respiratory infection, 3.6 million hospitalizations, and 26,300 in‐hospital deaths in 2019.^13^ After the outbreak of COVID‐19, the detection rate of HRSV has declined to varying degrees globally. In the United Kingdom, laboratory‐confirmed HRSV cases among children younger than 5 years of age experienced a drastic reduction of 99.5% in the winter of 2020–2021 compared with the predicted value.^14^ In a Brazilian prospective cohort study, HRSV was not detected in any of the 298 children with respiratory symptoms from May to August 2020.^15^ In Wuhan, China, HRSV decreased sharply in the short term after the COVID‐19 outbreak, with the detection rate declining from 9.4% to 2.8% (*P* < 0.001).^16^ Comparably low HRSV prevalence in children during COVID‐19 has also been found in other Chinese cities, such as Suzhou (4.91%),^17^ Shanghai (4.55%),^18^ and Beijing (3.15%).^19^ However, as NPIs were gradually relaxed, an unprecedented rebound of HRSV occurred in some regions, and the peak season has changed from winter and spring to summer. According to the HRSV National Trend Report released by the US Centers for Disease Control and Prevention (CDC), the positive rate of HRSV infection increased from 1.8% in March 2021 to 26% in June 2021.^20^ In Western Australia, Foley et al. reported a noteworthy surge in HRSV cases, starting in September 2020, above the average seasonal peak recorded from 2012 to 2019.^21^ A similar HRSV resurgence has been observed in many other locations in northern and southern hemispheres (South Africa, New Zealand, the United States, China, and Japan).^16^, ^22^ Moreover, it appeared that the median age of HRSV‐infected children was older than the pre‐COVID‐19 period, but there were no significant differences in the length of hospitalization or severity of the disease.^23^, ^24^

### 3.2 Influenza virus

During COVID‐19, large‐scale NPIs have led to the early end of the 2019/2020 influenza season, and influenza virus (IFV) activity almost disappeared throughout 2020. In Australia, between March and September 2020, despite extensive testing, the number of influenza cases decreased by 95.3% compared to the average reported cases for the same period from 2015 to 2019.^25^ A national surveillance study in Canada found that during the 2020/2021 influenza season, IFV‐A and IFV‐B detection rates dropped to 0.15% and 0.28% of the pre‐pandemic levels, respectively.^26^ The phenomenon that influenza activity has been extremely suppressed was also observed in China, Japan, South Korea, Thailand, and other countries.^17^, ^27^, ^28^, ^29^, ^30^ However, as the level of global NPIs has gradually been downgraded, influenza has regained its epidemiological characteristics, and outbreaks have occurred in some areas. In Shanghai, the peak IFV activity disappeared completely in 2020 and 2021, but it reappeared in the summer of 2022, and detection rates were significantly higher than those predicted by the model without NPIs.^31^ According to the weekly surveillance report of the National Influenza Center of China, 794 influenza‐like illness outbreaks (10 or more cases) were reported during the 14th to the 27th week of 2022 (from April 4 to July 10, 2022), mainly caused by H3N2.^32^

### 3.3 Human adenovirus

Human adenovirus (HADV) is a nonenveloped double‐stranded DNA virus, which is one of the common pathogens of lower RTIs in infants and young children. It is also an important viral pathogen causing severe pneumonia in children and may leave behind complications such as bronchiolitis obliterans and bronchiectasis.^33^ Epidemiological trends in HADV were inconsistent across regions during the pandemic, but a decrease in HADV activity was still observed in most regions. In China, the detection rate of HADV decreased significantly compared with that of pre‐COVID‐19 in Guangzhou (2.68%),^34^ Shenzhen (2.37%),^35^ Suzhou (0.33%),^17^ and Beijing (1.41%).^36^ However, in Hangzhou, the positive rate of HADV after the response to a major public health emergency in early 2020 was 4.52%, higher than that in January 2020 (2.92%) before the response.^30^ Trenholme et al. found that despite the significant decline in HRSV and IFV activity observed in New Zealand from January to August 2020, the prevalence of HADV remained similar to previous years.^37^

### 3.4 Human rhinovirus

Human rhinovirus (HRV) is a nonenveloped single‐stranded RNA virus with more than 160 serotypes that typically causes upper RTIs. Unlike most respiratory viruses, HRV appears to exhibit resilience during the COVID‐19 pandemic and continues to circulate actively. Several studies have reported an increase in detection of HRV during the pandemic, and in some areas, it has even become the most predominant viral pathogen for childhood RTI. A Japanese study found that respiratory viruses were significantly constrained in all age groups during COVID‐19 compared with the pre‐pandemic period, while HRV increased significantly in children under 10 years of age.^38^ In Germany, between November 2020 and April 2021, the detection rate of HRV in children with RTIs was as high as 48.8%.^39^ In Beijing, China, the positive rate of HRV in children with RTIs increased sharply from June 2020 (13.77%) and reached a peak in August (37.25%), which was significantly higher than that in the same period of 2017–2019.^40^ A study from Shanghai showed that HRV accounted for 52.5% of the total number of positive viral cases in children with RTI in 2020, with HRV‐A being the predominant subtype.^41^

### 3.5 Human metapneumovirus

Human metapneumovirus (HMPV) is an enveloped, single‐stranded, negative‐sense RNA virus known to cause both upper and lower RTIs in people of all ages. Among children under 5 years of age, the global prevalence of HMPV infection ranges from 1.1% to 86%.^42^ In 2018, there was an estimated 14.2 million HMPV‐associated acute lower respiratory infection cases in this age group globally.^43^ During COVID‐19, similar to RSV and IFV, HMPV exhibited a decline in activity when NPIs were strictly in place and a resurgence when NPIs were lifted. In 2020, the prevalence of HMPV notably decreased compared with pre‐pandemic years in China, Japan, Israel, Canada, and Australia.^38^, ^44^, ^45^, ^46^, ^47^ Conversely, in 2021, HMPV resurgence was observed, with Australia reporting a greater than fourfold increase in incidence among children aged 1–4 years.^48^, ^49^ According to the US CDC, in March 2023, the percentage of positive tests for HMPV spiked to 19.6% for antigen tests and 10.9% for polymerase chain reaction tests, surpassing RSV and SARS‐CoV‐2 levels during the same period.^50^

## 4 POSSIBLE MECHANISMS OF CHANGES IN THE VIRUS SPECTRUM DURING THE COVID‐19 PANDEMIC

Based on the above findings, NPIs targeting SARS‐CoV‐2 appear to be the primary cause of the decline of most seasonal respiratory viruses during the pandemic. NPIs are the first public health responses to infectious diseases, and it is implemented at three main levels: individual level (such as hand washing, mask wearing, and cough etiquette), social level (such as quarantine of exposed people and school and border closures) and environmental level (such as surface and objection disinfection). The mode of transmission of respiratory viruses mainly includes aerosol, droplet, and direct or indirect contact transmission and is also affected by many factors such as virus stability, environment, and host factors.^51^ However, NPIs have different effects on different pathogens. For example, during COVID‐19, detection rates of HRV and HADV have remained relatively stable or even exhibited an increasing trend compared to the pre‐pandemic period, which may be related to their absence of a viral envelope, insensitivity to lipophilic disinfectants, and virus particle size and the number of serotypes. In addition, a decrease in healthcare‐seeking behavior during the pandemic may be another possible cause, especially for children with mild symptoms.

Virus–virus interaction refers to the phenomenon that the infection process of one virus may be influenced by previous or simultaneous infection with another virus, and infection of the first virus can enhance or reduce infection and replication of the second virus, resulting in positive (additive or synergistic) or negative (antagonistic) interactions.^52^ Viral interference, usually referring to negative interactions, has been proposed since the 1960s, meaning that infection by a first virus causes cells or tissues to become resistant to infection by a second, unrelated virus.^53^ The mechanism of viral interference may be due to the interfering viruses blocking or reducing cell surface receptors and competing for cell resources and inducing a transient interferon (IFN) response allowing the host to acquire temporary nonspecific immunity. Nickbakhsh et al. showed that viral interference may be responsible for the decrease in HRV infection during the influenza season and that this interference may arise through an IFN‐mediated mechanism.^54^ Correspondingly, Wu et al. found in vitro experiments in differentiated airway epithelial cells in which HRV infection induced IFN‐stimulated gene expression and prevented IFV‐A infection, supporting the notion that HRV disrupted the 2009 European influenza pandemic.^55^ Other viral interferences reported in the literature exist in IFV‐A and HRSV,^56^ HRSV and HMPV,^57^ HRSV and HRV,^58^, ^59^ etc. However, little is known about how SARS‐CoV‐2 interacts with other respiratory viruses. It has been suggested that in animal models, HRV infection of human bronchial epithelial cells can impede SARS‐CoV‐2 replication by triggering the induction of multiple IFN‐stimulated gene expressions.^60^ Fage et al. suggested a negative correlation between IFV‐A (H1N1) pdm09 and SARS‐CoV‐2 in human nasal epithelial cells as well.^61^ Further exploration of the potential mechanisms of virus–virus interactions, particularly the interaction of SARS‐CoV‐2 with seasonal respiratory viruses, is crucial to the prediction of viral epidemic patterns. In addition, clarifying mechanisms of viral interference may also provide new ideas for clinical treatment.

By the end of 2022, global NPIs had been generally downgraded, and in Europe and the United States there is a collision of COVID‐19, influenza, and HRSV cases, which was called a “tripledemic.” A surge of pediatric cases in a short period has caused severe medical crowding and widespread concern among pediatricians.^62^, ^63^ There are various hypotheses about the causes of this phenomenon. The concept of “immunity debt” has been proposed by French scholars, referring that the lack of immune stimulation due to reduced exposure to pathogens, which increases the proportion of susceptible populations and decreases herd immunity, and may have a negative impact when pandemics are controlled and NPIs are lifted.^64^ It has also been suggested that the surge in respiratory virus infections after the pandemic was due to a large number of children experiencing delayed infections at the same time. Some scientists also hypothesized that the surge may be the result of compromised immune function caused by SARS‐CoV‐2 infection, making people more susceptible to other infections. In addition, it was also found that the median age of the surge in HRSV cases was older than that in the pre‐pandemic era, which may be attributed to the expansion of the RSV‐naive cohort as a result of strict NPIs, with some children who would have been infected with RSV a year or two ago being first exposed and infected with RSV only after NPIs were lifted.

## 5 REFLECTIONS ON THE DIAGNOSIS AND TREATMENT OF RTIs IN CHILDREN IN THE POST‐PANDEMIC ERA

On January 8, 2023, China announced the change of the name of “novel coronavirus pneumonia” to “novel coronavirus infection” under Category B management. However, as the virus continues to mutate, COVID‐19 is still ongoing worldwide, and our recognition of SARS‐CoV‐2 is still limited. In the post‐pandemic era, long COVID‐19 has become a public health concern for clinicians and researchers. Long COVID‐19 in children usually appears 12 weeks after initial SARS‐CoV‐2 infection, with an incidence of 1.6%–70%, and can involve multiple systems and children of all ages.^65^ More high‐quality, controlled studies with large sample sizes are urgently needed to further elucidate the characteristics and mechanisms of long COVID‐19 in children. In addition, it was found that the prevalence of SARS‐CoV‐2 coinfection with seasonal respiratory viruses in children was 9.39%, which is higher than that observed in adults (3.51%).^66^ In particular, IFV, HRSV, and HADV are the most commonly observed coinfecting viruses. Coinfection significantly increases the risk of severe outcomes such as respiratory distress, the need for invasive mechanical ventilation, and death.^66^, ^67^, ^68^ Therefore, in the post‐pandemic era, it is crucial to address how SARS‐CoV‐2 and seasonal respiratory virus coinfections impact the severity of childhood RTIs. Nowadays, as SARS‐CoV‐2 has become less virulent, COVID‐19 may gradually evolve into a seasonal and regional epidemic requiring regular vaccinations. At present, COVID‐19 vaccination in China primarily focuses on children over 3 years of age. Close attention should be given to the progress in the development of COVID‐19 vaccines, expanding the age range for vaccination, and proactively implementing vaccination prior to the arrival of the epidemic season to reduce risks of serious illness, death, and long‐term sequelae.

## 6 SUMMARY

During the COVID‐19 pandemic, the spectrum of respiratory diseases and respiratory viruses in children has undergone important changes. The widespread implementation of NPIs may be the primary contributing factor to the decline in most respiratory pathogens. However, the relaxation of NPIs has also led to a series of public health issues worthy of attention, including virus resurgence. In the post‐pandemic era, it remains crucial to continue monitoring the epidemiological trends of SARS‐CoV‐2 and other respiratory viruses, strengthening our understanding of long COVID‐19, coinfections involving SARS‐CoV‐2 and other viruses, and the effectiveness and impact of COVID‐19 vaccines. Furthermore, conducting in‐depth studies on the mechanisms of virus–virus interactions is essential. Altogether, these research endeavors will help accumulate valuable knowledge and enhance our confidence in responding to respiratory virus outbreaks that may reemerge in the future.

## AUTHOR CONTRIBUTIONS

Not applicable.

## CONFLICT OF INTEREST STATEMENT

Prof. Enmei Liu is the Deputy Editor‐in‐Chief. To minimize bias, she was excluded from all editorial decision‐making related to the acceptance of this article for publication. The remaining authors declare no conflict of interest.

## ETHICS STATEMENT

Not applicable.

## Data Availability

Data sharing is not applicable to this article as no new data were created or analyzed in this study.
